# Experimental Determination of the Threshold Dose for Bifidogenic Activity of Dietary 1-Kestose in Rats

**DOI:** 10.3390/foods9010004

**Published:** 2019-12-19

**Authors:** Ayako Watanabe, Yoshihiro Kadota, Hijiri Yokoyama, Shunya Tsuruda, Rina Kamio, Takumi Tochio, Yoshiharu Shimomura, Yasuyuki Kitaura

**Affiliations:** 1 Laboratory of Nutritional Biochemistry, Department of Applied Biosciences, Graduate School of Bioagricultural Sciences, Nagoya University, Nagoya 464-8601, Japan; watanabe.ayako@i.mbox.nagoya-u.ac.jp (A.W.); emk1218_maple@yahoo.co.jp (H.Y.); tsuruda.shunya@a.mbox.nagoya-u.ac.jp (S.T.); mihari.rina@outlook.jp (R.K.); 2 B Food Science Co., Ltd., Chita, Aichi 478-0046, Japan; y-kadota@bfsci.co.jp (Y.K.); t-tochio@bfsci.co.jp (T.T.); 3 Department of Food and Nutritional Sciences, College of Bioscience and Biotechnology, Chubu University, Kasugai, Aichi 487-8501, Japan; shimomura@isc.chubu.ac.jp

**Keywords:** prebiotics, 1-kestose, *Bifidobacterium*

## Abstract

1-Kestose is a non-digestible oligosaccharide consisting of glucose linked to two fructose units. While 1-kestose is not digested in the small intestine of mammals, it is fermented in the ceca and colon, where the growth of bifidobacteria is promoted. In the present study, we assessed the threshold dose of dietary 1-kestose that increased cecal bifidobacterial levels in rats. Rats were fed experimental diets containing 0% to 0.3% 1-kestose for four weeks. The levels of the genus *Bifidobacterium* and total gut bacteria were significantly increased in cecal samples of rats fed the 0.3% 1-kestose diet. Further, a significant correlation between the dose of 1-kestose and the levels of cecal *Bifidobacterium* and total gut bacteria was observed. The minimum dose of dietary 1-kestose to induce significant bifidogenic activity in rats was 0.3% by weight in the diet.

## 1. Introduction

Fructooligosaccharides (FOSs) have been recognized as prebiotics for their capability to modulate composition and/or activity of the gut microbiota for decades [1]. One such gut microbe is members of the genus *Bifidobacterium*, whose health benefits to the human host are well studied, ranging from improved constipation to colorectal cancer prevention and/or treatment (reviewed in [2]). FOSs are defined as a mixture of 1-kestose, nystose and 1^F^-ß-fructofuranosylnystose oligosaccharides [3]. 1-Kestose is the smallest FOS component, and consists of a single unit of fructose linked to another fructose unit by ß-glucosidic bonds carrying a single glucosyl unit as in sucrose. Due to its chemical structure and water solubility, 1-kestose is thought to be readily utilized by the gut microbiota. The daily administration of 1-kestose alone to gnotobiotic mice resulted in higher bifidobacterial numbers compared to the group administered FOSs [4]. The bifidogenic activity was significantly stronger with 1-kestose than nystose and FOSs in vitro [5]. In our previous study, we demonstrated that 1-kestose showed a dose-dependent relationship with bifidogenic activity in rats [6]. Since 1-kestose naturally occurs in several foods, such as banana, peach, artichoke, onion, barley and wheat [3], and only trace levels of 1-kesotse are consumed in the regular diet, its ability to exert prebiotic effects has drawn attention to its use as a supplement to a regular diet.

Currently, while the prebiotic activities of FOSs, galactooligosaccharides and inulin-type fructans have been widely tested (dose-dependent effects or threshold doses in healthy subjects [7,8,9]), the optimal dose of 1-kestose has not been adequately established. Since our previous finding confirmed that a 0.5% 1-kestose dietary supplementation was sufficient to increase the population of *Bifidobacterium* in rat cecal contents [6], this study investigated the minimum effective dose of 1-kestose to modulate beneficial microbes in rats.

## 2. Materials and Methods

1-Kestose (purified >98%) was provided by B Food Science Co., Ltd. (Chita, Aichi, Japan). The pellet form AIN-93G diet and the AIN-93G-based experimental diets were purchased from CLEA Japan (Tokyo, Japan). Equivalent amount of sucrose (0.1%, 0.2% or 0.3%) in AIN-93G were replaced with 1-kestose in the experimental diets (Supplementary Table S1) [6].

Eight-week-old male Sprague–Dawley (SD) rats (Japan SLC, Hamamatsu, Shizuoka, Japan) were housed individually in cages with a 12 h light/dark cycle at a controlled temperature (23 ± 1 °C) and given free access to food and water. Prior to feeding the experimental diet, the animals were maintained on a chow diet for 1 week. Thereafter, the rats were randomly allocated to 4 groups as control, 0.1% 1-kestose diet (KES), 0.2% KES and 0.3% KES (*n* = 8 per group), and fed the AIN-93G-based experimental diet containing 0%, 0.1%, 0.2% or 0.3% 1-kestose for 4 weeks. Food intake and body weight were recorded weekly. On the final day of the experiment, the rats were anesthetized with isoflurane, and blood, ceca and cecal contents were collected. The pH of the cecal contents was measured. Tissues were stored at −80 °C for later analyses. The study was approved by the Animal Care Committee of the Graduate School of Bioagricultural Sciences, Nagoya University.

Measurements of cecal microbiota populations were conducted using quantitative real-time PCR (qPCR) at Technosuruga Laboratory Co., Ltd. (Shizuoka, Japan). Details of the analyses were described previously [6]. Primers sequences for 16S rRNA analysis are shown in Supplementary Table S2 [10,11,12].

**Figure 1:**
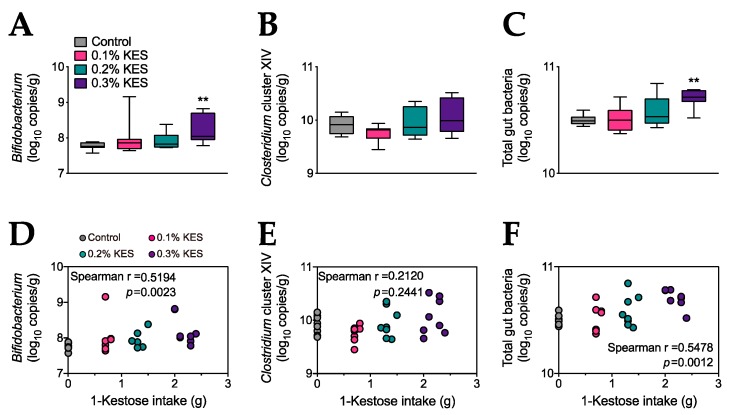
Supplementation with 1-kestose increases populations of *Bifidobacterium* and total gut bacteria. Populations of *Bifidobacterium* (**A**), *Clostridium* cluster XIV (XIVa and XIVb) (**B**) and total gut bacteria (**C**) were measured using qPCR (*n* = 8 per group). Spearman’s correlations between 1-kestose intake and either *Bifidobacterium* (**D**), *Clostridium* cluster XIV (**E**) or total gut bacteria (**F**) were estimated (*n* = 8 per group). Significant differences between the control and KES groups were analyzed using the Kruskal–Wallis test followed by Dunn’s multiple comparison test, ** *p* < 0.01. (**D**–**F**) The symbols represent individual values for 1-kestose intake and numbers of each bacteria. r and *p* values were determined using non-parametric Spearman’s correlations. KES, 1-kestose diet.

The data are presented as the mean ± SEM (Table 1 and Table 2), box-and-whisker plots where a range bar shows the median and interquartile range of the data with whiskers extended to minimum and maximum values (Figure 1A–C), as well as individual dot plots (Figure 1D–F). The data were analyzed using GraphPad Prism version 8.2.1 (GraphPad Software, San Diego, CA, USA). Multiple comparison tests were performed according to the data distribution, being either a Dunnett’s multiple comparison test (parametric test) or the Kruskal–Wallis test (nonparametric test) followed by a Dunn’s multiple comparison test. For correlations, Spearman’s correlation coefficient by rank was applied. A *p* <  0.05 was considered significant.

## 3. Results and Discussion

Body weight and food intake were not significantly affected by 1-kestose supplementation in all experimental groups (Table 1). Body weights were slightly higher in the 0.1% KES group than in the other groups; however, values among the four groups were not significantly different. Total 1-kestose intake in the four groups was confirmed to be dose-dependent according to the treatment groups (Table 1). 

The weights of the colon and cecum were slightly affected by 1-kestose supplementation; however, the differences among the four groups were not significant (Table 2). The pH of the cecum tended to be lower in the 1-kestose groups than in the control group, and was mildly acidified in the 0.3% KES group (Table 2).

Next, we assessed the effects of 1-kestose supplementation on the gut microbiota using 16S rRNA gene sequence analysis. The level of *Bifidobacterium* was significantly greater in the 0.3% KES group than in the control (Figure 1A). *Clostridium* cluster XIV (XIVa and XIVb), which includes short-chain fatty acid (SCFA) producers [13], did not show significant differences among the four groups (Figure 1B). Total bacterial levels appeared to increase in a 1-kestose dose-dependent manner, and levels were significantly greater in the 0.3% KES group than the control (Figure 1C). The levels of *Bifidobacterium* (Figure 1D) and total gut bacteria (Figure 1F), but not *Clostridium* cluster XIV (Figure 1E), were positively correlated with 1-kestose intake. 

To the best of our knowledge, the present study is the first to assess the minimum dose effects of 1-kestose supplementation in rats. Our analyses demonstrated that dietary supplementation of 1-kestose significantly increased the levels of *Bifidobacterium* and total gut bacteria in the cecal contents of rats fed 0.3% KES, and the levels of *Bifidobacterium* and total gut bacteria were positively correlated with the intake of 1-kestose, indicating that there is a threshold of dietary 1-kesotse to produce bifidogenic activity. 

On the other hand, no alterations in *Clostridium* cluster XIV were observed with 0.3% 1-kestose dietary supplementation. Carbohydrates such as non-digestible oligosaccharides (e.g., 1-kestose) are preferentially fermented by gut microbiota belonging to genera such as *Bifidobacterium* and *Lactobacillus* [9]. In these genera, lactate is produced as a major product of fermentation and can be utilized by other bacterial species to produce SCFAs [14,15]. *Clostridium* cluster XIV is a species capable of producing SCFAs by metabolic cross-feeding [16]. Therefore, our results suggest that dietary supplementation with 0.3% 1-kestose for 4 weeks may not be sufficient to increase *Clostridium* cluster XIV levels in the cecal contents of rats.

The 1-kestose intake in 0.3% KES was 0.16 g/day/kg body weight (Table 1), indicating that the dose of 0.3% 1-kestose is roughly equivalent to 10 g/day for humans of around 60 kg body weight. Although we cannot apply the dose to humans directly, the dose is at a comparable level to that in human studies with inulin-type fructans [7] and FOSs [8]. Further studies are required to determine the minimum effective dose of 1-kestose as well as health benefits such as improved constipation in humans. 

## Figures and Tables

**Table 1:** Body weight, food intake and 1-kestose intake ^1^.

Item	Control			0.1% KES			0.2% KES			0.3% KES		
Body weight (g)	455.8	±	8.2	481.6	±	7.8	455.6	±	6.1	466.3	±	7.9
Food intake (g/day) ^2^	23.2	±	0.6	25.4	±	0.6	23.5	±	0.8	25.5	±	0.7
1-Kestose intake (g) ^3^	0.00	±	0.0	0.72	±	0.02	1.34	±	0.04	2.18	±	0.05
1-Kestose intake (g/day/kg BW) ^4^	0.00	±	0.00	0.05	±	0.00	0.10	±	0.00	0.16	±	0.00

^1^ Values represent means ± SEM, *n* = 8 per group. ^2^ Food intake is the average of the 4 weeks of the experimental period. ^3^ 1-Kestose intake is the total of the 4 weeks of the experimental period. ^4^ 1-Kestose intake is estimated using the 4-week average 1-kestose intake divided by a kg rat body weight. KES, 1-kestose diet; SEM, standard error of the mean.

**Table 2:** Colon and cecum weights and cecum pH ^1,2^.

Item	Control			0.1% KES			0.2% KES			0.3% KES		
Colon (g)	0.894	±	0.037	0.920	±	0.046	0.940	±	0.030	0.948	±	0.036
Cecum (g)	0.804	±	0.027	0.922	±	0.041	0.877	±	0.037	0.897	±	0.032
Cecal pH	7.1			6.9			7.0			6.5		
	(6.7	−	7.2)	(6.7	−	7.2)	(6.5	−	7.3)	(6.4	−	7.1)

^1^ Values of colon and cecum weights represent means ± SEM, *n* = 8 per group. ^2^ Values of cecum pH represent the median with interquartile range in parentheses. KES, 1-kestose diet; SEM, standard error of the mean.

## Supplementary Materials

The following are available online at https://www.mdpi.com/2304-8158/9/1/4/s1, Table S1: Experimental diets. Table S2: Primer sequences.

## References

[1] Bindels L.B., Delzenne N.M., Cani P.D., Walter J. (2015). Towards a more comprehensive concept for prebiotics. Nat. Rev. Gastroenterol. Hepatol..

[2] O’Callaghan A., van Sinderen D. (2016). Bifidobacteria and their role as members of the human gut microbiota. Front. Microbiol..

[3] Campbell J.M., Bauer L.L., Fahey G.C., Hogarth A.J.C.L., Wolf B.W., Hunter D.E. (1997). Selected fructooligosaccharide (1-kestose, nystose, and 1f-β-fructofuranosylnystose) composition of foods and feeds. J. Agric. Food Chem..

[4] Suzuki N., Aiba Y., Takeda H., Fukumori Y., Koga Y. (2006). Superiority of 1-kestose, the smallest fructo-oligosaccharide, to a synthetic mixture of fructo-oligosaccharides in the selective stimulating activity on bifidobacteria. Biosci. Microflora.

[5] Ose R., Hirano K., Maeno S., Nakagawa J., Salminen S., Tochio T., Endo A. (2018). The ability of human intestinal anaerobes to metabolize different oligosaccharides: Novel means for microbiota modulation?. Anaerobe.

[6] Tochio T., Kitaura Y., Nakamura S., Sugawa C., Takahashi M., Endo A., Shimomura Y. (2016). An Alteration in the cecal microbiota composition by feeding of 1-kestose results in a marked increase in the cecal butyrate content in rats. PLoS ONE.

[7] Vandeputte D., Falony G., Vieira-Silva S., Wang J., Sailer M., Theis S., Verbeke K., Raes J. (2017). Prebiotic inulin-type fructans induce specific changes in the human gut microbiota. Gut.

[8] Bouhnik Y., Vahedi K., Achour L., Attar A., Salfati J., Pochart P., Marteau P., Flourie B., Bornet F., Rambaud J.C. (1999). Short-chain fructo-oligosaccharide administration dose-dependently increases fecal bifidobacteria in healthy humans. J. Nutr..

[9] Roberfroid M., Gibson G.R., Hoyles L., McCartney A.L., Rastall R., Rowland I., Wolvers D., Watzl B., Szajewska H., Stahl B. (2010). Prebiotic effects: Metabolic and health benefits. Br. J. Nutr..

[10] Gueimonde M., Tölkkö S., Korpimäki T., Salminen S. (2004). New real-time quantitative PCR procedure for quantification of bifidobacteria in human fecal samples. Appl. Environ. Microbiol..

[11] Song Y., Liu C., Finegold S.M. (2004). Real-time PCR quantitation of clostridia in feces of autistic children. Appl. Environ. Microbiol..

[12] Muyzer G., De Waal E.C., Uitterlinden A.G. (1993). Profiling of complex microbial populations by denaturing gradient gel electrophoresis analysis of polymerase chain reaction-amplified genes coding for 16S rRNA. Appl. Environ. Microbiol..

[13] Barcenilla A., Pryde S.E., Martin J.C., Duncan S.H., Stewart C.S., Henderson C., Flint H.J. (2000). Phylogenetic relationships of butyrate-producing bacteria from the human gut. Appl. Environ. Microbiol..

[14] Bourriaud C., Robins R.J., Martin L., Kozlowski F., Tenailleau E., Cherbut C., Michel C. (2005). Lactate is mainly fermented to butyrate by human intestinal microfloras but inter-individual variation is evident. J. Appl. Microbiol..

[15] Morrison D.J., Mackay W.G., Edwards C.A., Preston T., Dodson B., Weaver L.T. (2006). Butyrate production from oligofructose fermentation by the human faecal flora: What is the contribution of extracellular acetate and lactate?. Br. J. Nutr..

[16] Sarbini S.R., Rastall R.A. (2011). Prebiotics: Metabolism, structure, and function. Funct. Food Rev..

