# Supplementary material for: Experimental Determination of the Threshold Dose for Bifidogenic Activity of Dietary 1-Kestose in Rats
Source: Foods. 2019 Dec 19;9(1):4. doi: 10.3390/foods9010004 (PMC7022987; doi:10.3390/foods9010004)
Supplement: Supplementary file 1 [file foods-09-00004-s001.pdf]

Table S1. Experimental diets.

Ingredient	Control diet (0% 1-kestose)	1-Kestose diet (% 1-kestose)		
		0.1%	0.2%	0.3%
			(g/100 g diet)	
**Corn starch**	51.9486	51.9486	51.9486	51.9486
**α-Corn starch**	1	1	1	1
**Sucrose**	10	9.9	9.8	9.7
**Casein**	20	20	20	20
**Soybean oil**	7	7	7	7
**Cellulose**	5	5	5	5
**Mineral mix**	3.5	3.5	3.5	3.5
**Vitamin mix**	1	1	1	1
**L-Cystin**	0.3	0.3	0.3	0.3
**Choline bitartrate**	0.25	0.25	0.25	0.25
**tert-Butylhydroquinone**	0.0014	0.0014	0.0014	0.0014
**1-Kestose**	0	0.1	0.2	0.3
**Total**	100	100	100	100

Table S2. Primer sequences

Target bacteria		Oligonucleotide sequence (5' →3')	Reference
Bifidobacterium	F:	GATTCTGGCTCAGGATGAACGC	8
	R:	CTGATAGGACGCGACCCCAT	
Clostridium cluster XIVab	F:	GAWGAAGTATYTCGGTATGT	9
	R:	CTACGCWCCCTTTACAC	
16S rRNA	F1:	CCTACGGGAGGCAGCAG	10
	F2:	CGCCCGCCGCGCGCGGCGGGCGGGGCGG GGGCACGGGGGGCCTACGGGAGGCAGCAG	
	R:	ATTACCGCG GCTGCTGG	
